# Patient‐specific Instrumentation Affects Rotational Alignment of the Femoral Component in Total Knee Arthroplasty: A Prospective Randomized Controlled Trial

**DOI:** 10.1111/os.12420

**Published:** 2019-03-04

**Authors:** Davide Cucchi, Alessandra Menon, Alberto Aliprandi, Giulia Soncini, Beatrice Zanini, Vincenza Ragone, Riccardo Compagnoni, Paolo Ferrua, Chiara Fossati, Pietro Randelli

**Affiliations:** ^1^ Department of Orthopaedics and Trauma Surgery University Hospital Bonn Bonn Germany; ^2^ Laboratory of Applied Biomechanics, Department of Biomedical Sciences for Health University of Milan Monza Italy; ^3^ 1° Clinica Ortopedica, ASST Centro Specialistico Ortopedico Traumatologico Gaetano Pini‐CTO Monza Italy; ^4^ Zucchi Clinical Institutes Monza Italy; ^5^ IRCCS Policlinico San Donato San Donato Italy; ^6^ Department of Health Sciences “Amedeo Avogadro” University of Eastern Piedmont Novara Italy; ^7^ Department of Knee Surgery Azienda Socio Sanitaria Territoriale Centro Specialistico Ortopedico Traumatologico Gaetano Pini‐CTO Milan

**Keywords:** Alignment, Knee replacement, Patient Specific, Randomized, Rotation

## Abstract

**Objective:**

To evaluate whether patient‐specific instrumentation (PSI) improve the accuracy of femoral component rotational alignment with respect to conventionally‐implanted total knee arthroplasty (TKA).

**Methods:**

Twenty‐four patients were randomized to receive a TKA implanted with PSI or conventional instrumentation. Implant orientation was compared on Computed Tomography (CT). Surgical time, recuts, and component size variations from planning were recorded. Preoperative and postoperative Oxford knee score and visual analogue scale were compared to assess clinical outcomes.

**Results:**

Femoral components implanted with patient‐specific instrumentation were aligned with greater external rotation than those implanted with conventional instrumentation (*P* = 0.022). No significant differences were found in surgical times, number of recuts, and clinical outcomes. Surgeon modifications from the planned size were necessary in 58% of PSI cases.

**Conclusion:**

Femoral components implanted with PSI had greater external rotation than with conventional instrumentation. Surgeons must carefully evaluate component sizes when using PSI, both in planning and during surgery.

## Introduction

Total knee arthroplasty (TKA) is the treatment of choice for severe osteoarthritis of the knee. The demand for TKA has increased over the past 20 years, and it is expected to increase much more in the next few years. Therefore, there is a need for surgeons and implant companies to increase operating room efficiency and improve patient outcomes, while also lowering the cost.

Patient‐specific guides were first introduced in spine surgery in 1998 and at the beginning of the new century for TKA. Initial proponents of patient‐specific instrumentation (PSI) for TKA cite the following as potential advantages: decreases in surgical time, blood loss, number of instrument trays used, costs of the procedure, and planning time, and improvements in components’ alignment and clinical outcome^1^. PSI incorporates preoperative Computed Tomography (CT) or magnetic resonance imaging (MRI) to develop a preoperative plan, which includes the required amount of bony resections and a graphic representation of the implant positioning after surgery. Based on this planning, patient‐specific cutting guides are manufactured to fit patients’ specific bony anatomy. The precise preoperative plan is expected to help the surgeon avoid component malpositioning, which can negatively influence the results, causing aseptic loosening and unexplained pain^2^. Rotational alignment of the femoral component affects knee stability and kinematics,^3^, ^4^ and misalignment over 3° has been defined as an outlier from the desirable result based on previous evidence in the published literature^5^, ^6^, ^7^. Therefore, a new technology that could reduce the proportion of incorrectly aligned implants appears appealing. However, there is controversy in the available published literature on the ability of PSI to affect the implant positioning, with some studies showing increased accuracy of femoral rotation with the use of PSI^8^, ^9^, ^10^, ^11^ and others revealing no differences from conventional alignment techniques^12^, ^13^, ^14^, ^15^, ^16^, ^17^, ^18^, ^19^, ^20^.

This prospective randomized controlled clinical trial was designed to verify whether the use of PSI increases the accuracy of the rotational alignment of the TKA femoral component in comparison to a control group of conventionally‐implanted TKA, and to compare surgical and clinical performance between the two groups.

## Materials and Methods

### *Study design*

**Figure 1:**
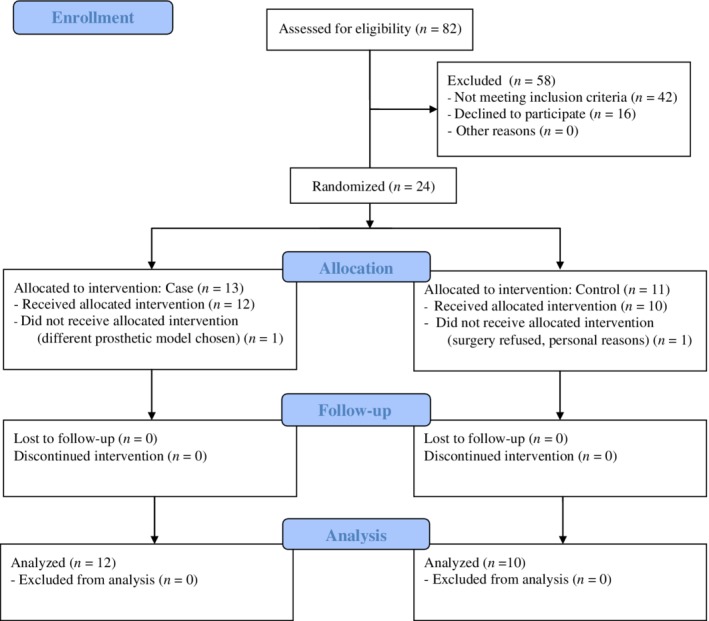
Flow diagram of the study.

The Consolidated Standards of Reporting Trials (CONSORT) statement guidelines were followed to perform this randomized controlled trial and to present the results. A flow diagram according to CONSORT guidelines illustrates the grouping and flow of patients in our clinical study (Fig. 1). Institutional review board approval was obtained prior to beginning this prospective study.

The primary goal of the present study was to evaluate whether a more precise rotational alignment of the femoral component to the surgical transepicondylar axis could be obtained using PSI. A power analysis revealed that, with a power of 80% and a significance level of 5% (two‐sided Student's *t*‐test), the minimum sample size to test a difference of 3° between the two groups was 18 patients (9 per group).

Secondary goals were to compare the two groups for number of recuts performed, ischemia time, total surgical time, and clinical results, and to analyze the surgeon's intraoperative modifications from the original planned component size in the PSI group.

Informed consent was obtained from all individual participants included in the study.

### *Enrolment, Preoperative Evaluations, and Planning*

A total of 24 patients with indication for total knee replacement and without any metal devices within 8 cm from knee articular surfaces or any fixed deformities greater than 15° in varus, valgus, flexion or tibial slope were prospectively enrolled by two investigators (D.C. and G.S.) between April 2013 and June 2014. All patients underwent clinical examination, and long leg and knee radiographs between 34 and 148 days prior to surgery. Block randomization was performed to allocate patients to control or treatment groups. A random allocation list, with 4 patients per block, was generated by an investigator (V.R.) neither involved in patient enrolment nor in data analysis, who concealed the sequence in sequentially numbered sealed envelopes until interventions were assigned. Patients allocated to the treatment group underwent a CT scan from the hip to the ankle, according to the manufacturer's instructions. For these patients, CT scan and morphometric data were sent to the manufacturer for instrument design and production. A “patient proposal,” containing the preoperative planning for the cutting guides, was then sent to the surgeon for careful examination and approval or requests of modification; every variation to the patient proposal was noted. Immediately prior to the surgery a new clinical examination was performed, blood samples were taken, and the patient was asked to complete the Oxford knee score (OKS) questionnaire^21^ and the visual analogue scale (VAS) evaluation tool.

### *Operative and Perioperative Procedures*

A cemented, posterior‐stabilized, mobile‐bearing prosthesis with patellar resurfacing (P.F.C. Sigma, DePuy International, St Anthony's Road, Leeds LS11 8DT, UK) was implanted using a medial parapatellar approach by the same senior surgeon (P.R.) in all patients. To verify the study hypothesis, a 0° rotation relative to the surgical transepicondylar axis^22^ was set as a rotation landmark for the femoral component for both conventionally‐implanted and PSI‐assisted prostheses. Conventional cutting instruments were used in the control group: an intramedullary femoral guide was set to align the component in 5° of valgus and an extramedullary tibial guide was set to align the component perpendicular to the mechanical axis in the coronal plane with 3° of posterior slope. The intraoperatively hand‐measured surgical transepicondylar axis was used as a reference for the femoral component rotational alignment and Akagi's line^23^ was used as a reference for the tibial component rotational alignment.

Trumatch PSI cutting guides (DePuy Orthopaedics, 700 Orthopaedic Drive Warsaw, IN 46581‐0988, USA) were used in the treatment group to perform proximal femur and distal tibial cuts, and to guide the axial positioning of the four‐in‐one cutting block, as indicated by the manufacturer's surgical guide. Accurate removal of all soft tissues in the area of guide supports was checked in all PSI surgeries to avoid guide misplacement. Coronal and sagittal orientation of the components was calculated using the Trumatch software from the preoperative CT scan. A 0° rotation relative to the surgical transepicondylar axis was set as a rotation landmark for the femoral component in the Trumatch software for the cutting guide draft design.

In all patients, the tourniquet was inflated before the incision and released before insert placement. Anesthetic and pain‐control medications, antithrombotic and antibiotic prophylaxes, and rehabilitation procedures were standardized according to the institution's internal protocols. During surgery, tourniquet time (from inflation to release), total surgical time (from incision to skin closure), femoral and tibial recuts needed, implant size, and complications of any kind were noted for all patients.

### *Postoperative Evaluation*

Two months after surgery, all patients underwent clinical examination and knee CT scans using a scatter reduction protocol and were asked to complete the OKS questionnaire and the VAS evaluation tool. The postoperative CT scans were independently analyzed with two‐decimal accuracy by two investigators (D.C. and G.S.) to measure the femoral component rotation to the surgical and clinical transepicondylar axis (TEA): these axes are defined as the line connecting the tip of the lateral epicondyle to the medial epicondylar sulcus (surgical TEA [s‐TEA], primary study goal) or to the medial epicondylar ridge (clinical TEA [c‐TEA]), as described by Berger *et al* 22.

### *Statistical Analysis*

Data were expressed as means ± standard deviation (SD). The differences between the two groups of patients for continuous variables were confirmed with an unpaired Student's *t*‐test or Mann–Whitney test according to the characteristics of the data distribution. The differences for categorical variables were tested with the χ^2^‐test or Fisher's exact test. The intraclass correlation coefficient was calculated to establish the agreement level between the two rates for the radiological measurements.

Statistical analysis (A.M.) was performed using GraphPad Prism v 6.0 software (GraphPad Software) and SPSS software (SPSS version 17, Chicago, IL, USA). For all analyses, the significance level was set at *P*‐value lower than 0.05.

## Results

Twenty‐two patients completed the follow‐up. In the treatment group, 1 patient refused intervention for personal reasons; 1 patient in the control group had a different prosthesis implanted and was, therefore, excluded. Patients’ demographics are reported in Table 1 and the main radiological and clinical results are in Table 2.

**Table 1:** Patients’ demographics

Group	Overall	Conventional	PSI	*P*‐value
Age	71.95	69.20	74.25	0.07
BMI	28.07	27.9	28.7	n.s.
F/M ratio	0.77/0.23	0.8/0.2	0.75/0.25	n.s.
L/R ratio	0.59/0.41	0.6/0.4	0.58/0.42	n.s.

BMI, body mass index; F/M, female/male; L/R, left/right; n.s., not significant; PSI, patient‐specific instrumentation.

**Table 2:** Summary of main radiological and clinical results (mean ± SD)

Group	Conventional	PSI	*P*‐value
ER to s‐TEA (°)	0.56 ± 2.47	2.88 ± 1.94	0.022
ER to c‐TEA (°)	−3.43 ± 2.73	−1.42 ± 1.69	0.047
Total surgical time (min)	84.60 ± 20.82	81,42 ± 16,64	0.694 (n.s.)
Ischemia time (min)	61.8 ± 17.85	62 ± 15.42	0.978 (n.s.)
ΔOKS (points)	+12.8 ± 8.73	+7.17 ± 10.50	0.192 (n.s.)
ΔVAS (mm)	−37.1 ± 20.0	−35.8 ± 27.4	0.904 (n.s.)

c‐TEA, clinical transepicondylar axis; Δ, delta; ER, external rotation; n.s., not significant; OKS, Oxford knee score; SD, standard deviation; s‐TEA, surgical transepicondylar axis; PSI, patient‐specific instrumentation; VAS, visual analogue scale.

**Figure 2:**
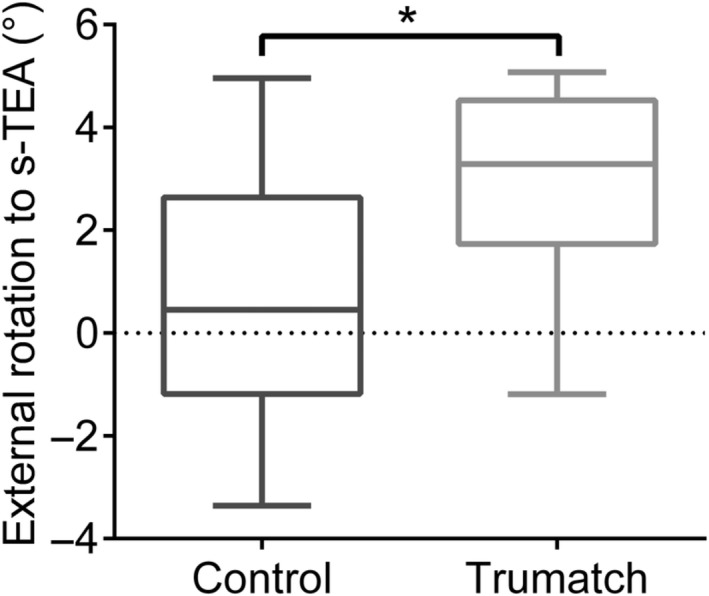
Component external rotation to the surgical transepicondylar axis (s‐TEA). Error bars show the mean ± SD. *P*‐values were calculated using Student's *t*‐test. *P*‐values are indicated: *P < 0.05, as compared to controls.

When comparing the two techniques, a more externally rotated femoral component, with reference to the s‐TEA, was observed in the PSI group (*P* = 0.022) (Fig. 2).

A similar difference was found when analyzing the femoral component rotation with reference to the c‐TEA (*P* = 0.047).

Excellent inter‐observer agreement was obtained for both series of measures (intraclass correlation coefficient for external rotation to s‐TEA: 0.903; to c‐TEA: 0.936). No significant difference between the number of outliers was found between the two groups.

The number of recuts performed, respectively, on the femoral and on the tibial side was 0 (0%) and 4 (40%) in the conventional group, and 1 (8%) and 3 (25%) in the PSI group. No significant difference was found between two groups.

**Figure 3:**
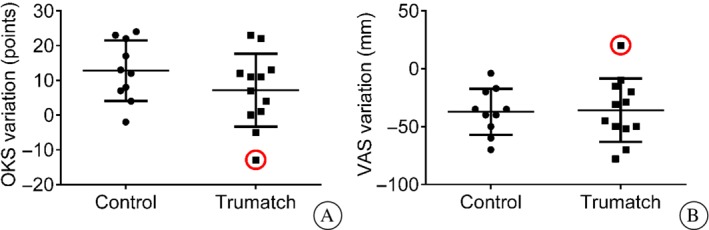
Comparison of clinical results 2 months after surgery: Oxford knee score (OKS) variation from preoperative level (A); visual analogue scale (VAS) variation (mm) from preoperative level (B). Note the outlier in the patient‐specific instrumentation group, corresponding to a case complicated by tibial guide breakage.

The analyses of ischemia time and total surgical time (Table 2), postoperative OKS gain (Fig. 3), and VAS reduction (Fig. 3) revealed no significant differences between the two groups.

In the PSI group, patient proposal was modified in 4 patients (33%): 1 femoral and 4 tibial components were upsized. Intraoperative deviations from planning were registered in 4 cases (33%): 1 femoral and 3 tibial components were upsized; 1 tibial component was downsized (21% of all implanted components). Overall, surgeon modifications from the original planned component size were necessary for 7 patients (58%). In 1 of these cases, the tibial component was upsized at the surgeon's first revision of the patient proposal and downsized intra‐operatively, thus returning to the originally suggested size. In 1 case, the external rotation of the femoral component was reduced by 1.5° to obtain optimal ligamentous balance.

In 1 case, the tibial cutting guide broke during the cut and conventional extramedullary alignment was used to complete the tibial cut; postoperative OKS was reduced by 13 points and VAS increased by 20 mm for this patient, in contrast with the trend of all other cases (Fig. 3). No other complications were observed.

## Discussion

**Figure 4:**
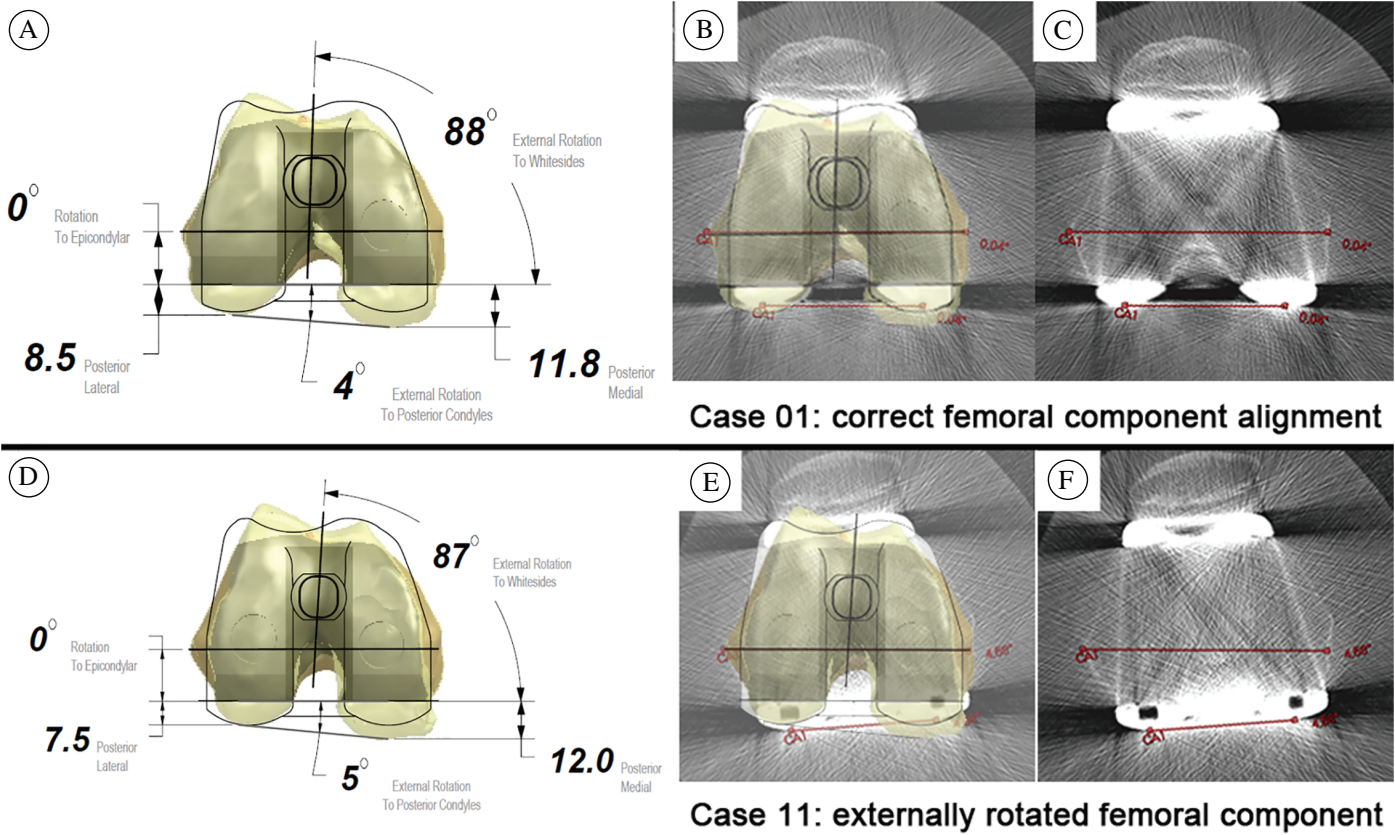
Preoperative planning (A, D), postoperative CT scans (C, F) and corresponding superimposition images (B, E) of femoral components in two cases: Case 01 (A–C): Correct alignment. Case 11 (D–F): Externally rotated component (4.48° mismatch from planning).

The main finding of our study was that PSI does not increase the accuracy of femoral component rotation in TKA. Indeed, a difference was found in favor of the conventional instrumentation group, with the PSI‐implanted femoral components being more externally rotated (Fig. 4).

Rotational alignment of the femoral component affects flexion stability, tibiofemoral and patellofemoral kinematics, and alignment in flexion^3^, ^4^.

Internal rotation of the femoral component has been associated with pain, stiffness, and instability^24^, ^25^. In contrast, excessive external rotation of the femoral component leads to symptomatic flexion instability, increased shear forces on the patella, and medial compartment overload in flexion^6^, ^26^.

Bell *et al*. identified internal rotation misalignment of the tibial and femoral components individually as well as the combined component rotation and component rotation mismatch to be factors in pain following TKA. External rotation of the component parameters was not identified to be a factor in painful TKA^2^. In our study, the difference in rotational alignment between the two groups was not combined with a difference in immediate postoperative clinical outcomes measured with OKS and VAS.

Patient‐specific instrumentation has not yet been clearly demonstrated as effective in increasing the accuracy or precision of femoral component rotation: several studies have investigated the effect of PSI on rotational alignment and, with a few exceptions^8^, ^27^, did not find statistical difference with respect to the outliers of femoral component rotation^14^, ^15^ and the accuracy in postoperative alignment of femoral rotation^12^, ^13^, ^14^, ^20^. Two recent meta‐analyses enrolled only 9 studies that reported femoral rotation as an outcome, and reached conflicting results: Thienpont *et al*. concluded that no differences with regard to the rotational alignment are to be expected in the axial plane with the use of PSI,^28^ whereas Huijbregts *et al*. calculated the femoral rotation to be 0.45° more accurate with PSI^9^.

Reduction in the number of outliers from target rotational femoral component alignment indicates increased precision: controversial results have been obtained regarding this outcome, with some study groups reporting significant differences in favor of PSI^5^, ^10^ and others not^6^, ^7^, ^29^. Our study could not identify any difference in the proportion of outliers, both when following the “stricter” (±2°) and “looser” (±3°) criteria to define outliers proposed in the literature.

The variance of distribution for postoperative rotational alignment indicates variability in planning and component positioning: lower variance indicates greater precision of the surgical system. Significantly greater variance of distribution was reported without PSI by one group^30^, results which could not be confirmed by our series.

We hence cannot recommend PSI as a reliable system to ensure that the femoral component is placed accurately in a predetermined rotational position in the axial plane. It should also be kept in mind that the definition of correct rotational alignment remains controversial; the surgical transepicondylar axis is considered an optimal reference^31^, but its identification is biased by a high inter‐observer and intra‐observer variability^32^. Therefore, we agree in considering it wiser not to rely systematically on a single reference axis or technique for every patient.

Patient‐specific instrumentation was proposed as a solution to reduce surgical time. Significantly shorter operative times were consistently observed when comparing PSI with computer‐assisted surgery^33^. However, conflicting results were obtained in recent meta‐analyses^12^, ^15^ and dedicated clinical trials when comparing PSI and conventional instrumentation, with only some authors reporting reduction in operative times using PSI^1^, ^12^. Our study was not adequately powered to detect any significant differences in tourniquet time and total surgical time.

Ease of use and reliability are key features for a surgical instrument. A theoretical advantage of an ideal PSI is the possibility of carefully planning the intervention outside the operating theatre, reducing then the need for multiple intraoperative controls or allowing less experienced surgeons to operate on a reliable guide designed by a senior consultant. However, in our experience, repeated evaluations and changes are needed both preoperatively (33%) and intraoperatively (58%). This is in agreement with published reports that highlight that significant changes in the technician plan were necessary to obtain an accurate preoperative plan and that intraoperative size changes were common when using PSI^6^, ^34^, ^35^.

A poor match between the preoperative plan and intraoperative observations may lead the surgeon to abandon the PSI guide and switch to a conventional procedure^6^, ^7^, ^14^. In our series, conversion from PSI to conventional instrumentation was necessary in only 1 case, after the breakage of a tibial guide during the cut. Although surgery was completed successfully, the OKS decreased and VAS increased 2 months after the intervention; OKS decrease indicates that patient expectations have not been achieved after primary TKA^21^. At a 20‐month follow‐up, the patient reported a persistent burning sensation in and around the knee, with a VAS of 60 mm (preoperative: 50 mm) and an OKS of 26 points (preoperative: 26 points).

From this preliminary experience, we hence cannot recommend this CT‐based PSI to inexperienced users and advise careful evaluation by an experienced surgeon in both the planning phase and the cutting guide positioning. Further improvements in PSI technology, with better cartilage recognition algorithms for CT‐based PSI and more dimensional accuracy of bone modeling for MRI‐based PSI, are awaited to overcome the present limits of this promising technology.

PSI remains a valid instrument to handle complex extra‐articular deformities or to address cases in which difficulty in intramedullary rod passage is present due to deformity, retained hardware, or pathological bone disease, and when it is necessary to reduce blood loss^36^.

The present study has some limitations. First, we did not use intraoperative surgical navigation in the control group but considered sufficient a postoperative CT evaluation to determine component position. However, conventional instrumentation is considered the standard of care and CT‐determined rotation has been demonstrated to correlate with the actual component rotation. Second, all surgeries were conducted by a high‐volume knee surgeon (200 implants/year); the results of this study may not be applicable to a less experienced or lower volume surgeon, especially in the PSI group, in which the surgeon's experience is critical in identifying imperfections in surgical planning or malpositioning of the PSI guides. We also acknowledge a bias for the introduction of a new implant system and the potential influence of the learning curve; to limit these confounders, the manufacturer provided technical support for every intervention and helped with the logistic workflow in the hospital. Moreover, the study population was composed of a relatively small number of elderly White patients, with a large female dominance and follow‐up was limited to 2 months; this should be considered prior to extrapolating the significant findings to the general population, and studies with a larger sample size are expected to confirm these preliminary findings. Finally, a single type of PSI was tested (Trumatch System for PFC Sigma, DePuy Orthopaedics, Warsaw, IN, USA); other systems may perform differently and these results may then not be representative for all different custom‐fit technologies available.

### *Conclusion*

Patient‐specific instrumentation did not increase the accuracy of femoral component rotation in TKA, with PSI‐ implanted femoral components being more externally rotated than those implanted with conventional instrumentation. No significant improvements in ischemia time, total surgical time, and clinical outcomes could be identified. Extreme care must be taken by surgeons using PSI when evaluating component sizes, both in the preoperative planning and during surgery.
